# EMAST Type of Microsatellite Instability—A Distinct Entity or Blurred Overlap between Stable and MSI Tumors

**DOI:** 10.3390/genes14071474

**Published:** 2023-07-19

**Authors:** Kristina Vuković Đerfi, Anamarija Salar, Tamara Cacev, Sanja Kapitanović

**Affiliations:** Laboratory for Personalized Medicine, Division of Molecular Medicine, Rudjer Boskovic Institute, Bijenicka cesta 54, 10000 Zagreb, Croatia; kvukovic@irb.hr (K.V.Đ.); asalar@irb.hr (A.S.); kapitan@irb.hr (S.K.)

**Keywords:** microsatellite instability, MSI, elevated microsatellite alterations at selected tetranucleotide repeats, EMAST, mismatch repair, MMR, MSH3, cancer, immunotherapy

## Abstract

Microsatellite instability (MSI) represents an accumulation of frameshifts in short tandem repeats, microsatellites, across the genome due to defective DNA mismatch repair (dMMR). MSI has been associated with distinct clinical, histological, and molecular features of tumors and has proven its prognostic and therapeutic value in different types of cancer. Recently, another type of microsatellite instability named elevated microsatellite alterations at selected tetranucleotide repeats (EMAST) has been reported across many different tumors. EMAST tumors have been associated with chronic inflammation, higher tumor stage, and poor prognosis. Nevertheless, the clinical significance of EMAST and its relation to MSI remains unclear. It has been proposed that EMAST arises as a result of isolated MSH3 dysfunction or as a secondary event in MSI tumors. Even though previous studies have associated EMAST with MSI-low phenotype in tumors, recent studies show a certain degree of overlap between EMAST and MSI-high tumors. However, even in stable tumors, (MSS) frameshifts in microsatellites can be detected as a purely stochastic event, raising the question of whether EMAST truly represents a distinct type of microsatellite instability. Moreover, a significant fraction of patients with MSI tumors do not respond to immunotherapy and it can be speculated that in these tumors, EMAST might act as a modifying factor.

## 1. Introduction

### 1.1. Genomic Instability as a Hallmark of Cancer

Sustained proliferative signaling, evasion of growth suppressors, resistance to cell death, replicative immortality, neoangiogenesis, and acquisition of invasive and metastatic properties are functional capabilities, hallmarks of cancer, which any given cell passing through the multistep process of malignant transformation has to acquire [1]. The main enabling forces that fuel these processes are genomic instability and chronic inflammation as they create a favorable milieu for promotion of genetic alterations, increased growth, proliferation, and survival of cancer cells [2]. Yet, as they act globally throughout tumorigenesis in intricate networks and often with apparently opposing effects depending upon the phase of tumor evolution, their exact influence on patients’ outcome and response to therapy still remains elusive. This is especially true for tumors of the gastrointestinal tract, in which both chronic inflammation as well as chromosomal and microsatellite instability play a significant role in their tumorigenesis. In comparison to the well-established form of microsatellite instability, known as MSI [3], elevated microsatellite alterations at selected tetranucleotide repeats (EMAST) is an emerging form of microsatellite instability and, as such, is still poorly understood. Both EMAST and MSI-H exhibit some distinct molecular and clinicopathological features; however, lack of consensus of what EMAST is and how it is tested has led to confusion and often opposing opinions on whether EMAST exists as a separate entity outside of the MSI context [4].

### 1.2. Two Types of Microsatellite Instability, MSI and EMAST

Microsatellite instability (MSI), as a type of genomic instability, has been identified in the early days of molecular oncology in both hereditary and sporadic pathways of tumorigenesis [5,6,7]. It is characterized by frameshifts in microsatellite sequences, i.e., insertions and deletions in short tandem nucleotide repeats due to impaired DNA mismatch repair (MMR). If the MMR system ceases doing its role in the cell due to dysfunction of any of its components, the mutational rate rises, causing the hypermutated genotype that provides ideal conditions for neoplastic transformation and progression [8].

The term MSI was first mentioned in 1993 in the context of genomic instability detected in the proximal colon cancer [9]. In the same year, the association between hereditary nonpolyposis colorectal cancer (HNPCC) and germline mutations in the *MSH2* gene was established [10,11]. Subsequent studies have identified germline mutations in other *MMR* genes, including *MLH1*, *PMS2*, and *MSH6* that also contribute to the development of HNPCC. Thus, identification of hereditary mutations in *MMR* genes led to the establishment of the genetic basis of MSI. Nowadays, the term Lynch syndrome is used instead of HNPCC to describe cancers linked to the inactivation of the *MMR* genes *MSH2*, *MLH1*, *MSH6*, and *PMS2* [12]. Due to the inherited MMR deficiency followed by a somatic mutational change during their lifetime, patients affected by this condition have an elevated risk of developing colorectal cancer (CRC) as well as other cancers affecting the gastrointestinal, reproductive, and urological tract [13,14].

Nonetheless, microsatellite instability can also occur in tumors without an inherited MMR deficiency. MSI in the case of sporadic carcinomas is predominantly caused by hypermethylation of the *MLH1* promoter, which in essence represents the silencing of the *MLH1* gene [15]. In addition to colon, rectum, and gastric adenocarcinomas, MSI was also reported in other tumor types, such as endometrial, ovarian, prostate cancer, and glioblastoma [16]. In the study examining the microsatellite instability across different tumor types, the prevalence of MSI had quite a wide range, from 31.4% in endometrial cancer to only 0.25% in glioblastoma multiforme. The highest MSI prevalence was reported in endometrial, colorectal, and gastric cancers, which are also tumors that can develop in the context of the Lynch syndrome. Interestingly, in thyroid carcinoma and some rare tumor types, MSI was not detected [17].

In addition to MSI, it is nowadays recognized that tumor cells can harbor another type of microsatellite instability named elevated microsatellite alterations at selected tetranucleotide repeats (EMAST). Although its existence was recognized as early as MSI, it was not considered as a separate and/or relevant form of genetic instability until recently, and thus, it still remains poorly understood [8,18]. Despite not being initially referred to as EMAST, this phenomenon has been reported in colorectal, gastric, skin, bladder, ovarian, and non-small cell lung cancer. Since EMAST was first recognized, a very wide range of its prevalence was reported (11% to 64%) [19]. Such a wide range can be a result of differences in the underlying tumor biology. However, it can also be a result of different EMAST definitions and a lack of consensus resulting in the usage of different markers and/or different thresholds. As EMAST was mostly investigated in CRC, Ranjbar et al. performed a meta-analysis, which included 4922 patients from 16 studies and the reported prevalence of EMAST in CRC was 33% [20]. But, when one looks more closely, studies included in this meta-analysis appear to be very heterogeneous. Most studies use the panel of five tetranucleotide markers MYCL1, D8S321, D9S242, D20S82, and D20S85, while the cut-offs differ. In most studies, tumors presenting with instability at two or more loci are considered as EMAST positive. The range of observed EMAST prevalence using these markers was between 33–46% in the US population, while studies performed in Asian populations had lower EMAST frequencies ranging from 11–22% [20].

Interestingly, two early studies in CRC patients from the United States, which are often overlooked since the term EMAST was not yet used, employ a completely different set of 10 tetranucleotides, resulting in EMAST frequencies of 16.5% and 13.8%, respectively [21,22]. Contrary to this, Haugen et al. report an EMAST frequency of 60% in sporadic colorectal cancers, which were analyzed using the most widely used set of markers (MYCL1, D20S82, D20S85, D8S321 and D9S242) with the addition of markers L17835 and D19S394. The tumor was considered EMAST positive when instability was present at more than one locus [23]. In later studies by Yamada et al., Garcia et al., and Koi et al., which also employed 5 + 2 tetranucleotide markers, high EMAST frequencies of 64%, 40% and 61% were reported, respectively [24,25,26]. These examples nicely illustrate how usage of different markers and thresholds can generate such vast differences in EMAST prevalence even within the same tumor type. A lack of consensus on EMAST testing and its overlapping features with MSI has led to different views on whether EMAST exists as a separate entity outside the MSI context.

Therefore, the aim of this review was to examine the existing scientific literature in an attempt to answer the question: Is EMAST a distinct type of microsatellite instability or blurred overlap between stable and MSI tumors?

### 1.3. DNA Mismatch Repair and Mechanisms Leading to Microsatellite Instability

In order to try to resolve this question, we have to examine the molecular processes leading to microsatellite instability. As it was established by early studies in hereditary cancer with MSI characteristics, defective DNA mismatch repair (MMR) lies at the core of microsatellite instability. MMR is a highly conserved biological mechanism that plays a role in postreplicative DNA repair. The initial event in mismatch repair is the heterodimerization of MSH2 protein with either MSH6 (forming the MutSα complex/heterodimer) or MSH3 (forming the MutSβ complex), followed by the recognition of an error in the DNA sequence. The MutSα complex recognizes single mismatched base pairs, as well as mono- and dinucleotide frameshift mutations, while MutSβ recognizes dinucleotide and longer frameshift mutations [27]. There is a functional overlap between MutSα and MutSβ in the recognition of dinucleotide repeats, but biochemical studies have shown that MutSβ has a slight preference for dinucleotide recognition [28]. After these complexes have detected an error in the DNA sequence, MLH1-PMS2 (forming the MutLα complex) enables targeted DNA repair via DNA polymerase δ. However, if the error within the sequence is significant, this will trigger cell death, thereby ensuring genome integrity [27].

Historically, the term microsatellite instability was used for frameshifts at any type of microsatellite loci consisting of mono- to five-nucleotide repeats or even longer. In 1997, a consensus on MSI detection was reached during the National Cancer Institute (NCI) workshop held in Bethesda, MD, USA, and though the existence of instability at tetranucleotide and longer repeats was recognized, it was not included in the standardized MSI detection panel. The “Bethesda panel” as it is often called, consists of two mononucleotides (BAT25 and BAT26) and three dinucleotide microsatellite repeats (D2S123, D5S346 and D17S250). Depending upon the number of microsatellite loci affected by instability, highly unstable tumors (MSI-H, if two or more loci of the panel are affected) and tumors with low levels of microsatellite instability (MSI-L, only one locus is affected) have commonly been defined as specific subgroups of MSI. Tumors without instability at any of the five microsatellite loci are classified as microsatellite stable (MSS) tumors [8]. After this consensual panel has been established, most studies investigating MSI focused only on molecular changes at mono- and dinucleotides. These efforts resulted in the current understanding that identifying the presence of MSI in tumors bears significant clinical implications, since patients diagnosed with high levels of MSI (MSI-H) have better prognosis and follow different therapeutic algorithms [29].

**Figure 1:**
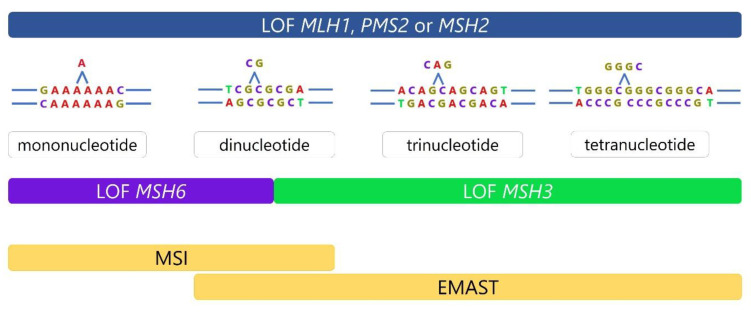
Different consequences of MMR defects. *MSH2*, *MLH1*, or *PMS2* loss of function (LOF) results in complete absence of MMR leading to instability at mono-, di-, and tetranucleotide repeats. On the other hand, defects in *MSH6* or *MSH3* are more specific. Dysfunctional MSH6 is associated with frameshifts at mono- and dinucleotide sequences, while dysfunction in MSH3 results in frameshifts at dinucleotide or longer repeats and absence of instability at mononucleotide repeats.

But, if we look more closely at possible consequences of loss of function of each of the main components of the MMR system, it becomes obvious that they do not have the same consequences. For instance, in the case of MSH2, MLH1, or PMS2 loss of function (LOF), the MMR is completely absent, leading to instability at mono-, di-, and tetranucleotide microsatellite repeats [30]. On the other hand, in the case of non-functional MSH6 or MSH3 proteins, a specific type of instability is present, associated with their distinct roles in mismatch repair [18,31]. Non-functional MSH6 proteins are associated with instability at mono- and dinucleotide microsatellite sequences, while in the case of non-functional MSH3 proteins, instability is present in dinucleotide or longer repeats, but not in mononucleotide sequences (Figure 1) [4].

### 1.4. Dual Etiology of EMAST—Two Sides of the Same Coin

In the context of MMR, reduction, or complete loss of nuclear MSH3 expression has been associated with the presence of EMAST in tumor cells. Haugen et al. first experimentally linked the MSH3 with EMAST by showing the loss of MSH3 expression in sporadic CRCs with EMAST, which was further corroborated by in vitro results showing that MLH1- and MSH3-deficient CRC cell lines exhibit instability at several tetranucleotide loci [23]. Furthermore, it has been proposed that reduced and heterogeneous expression of nuclear MSH3, as sometimes seen in sporadic EMAST CRCs, is not due to *MSH3* mutation or epigenetic inactivation, but rather is caused by a change in its cellular localization. Specifically, MSH3 shifts from the nucleus, where it plays a role in DNA surveillance and repair, to the cytosol [31,32].

**Figure 2:**
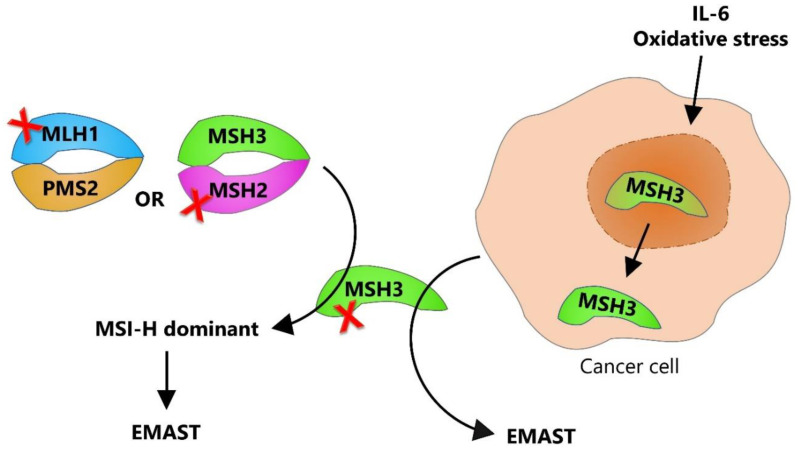
Dual etiology of EMAST. EMAST can arise by two different mechanisms: one associated with pre-existing inactivation of DNA MMR (mostly due to MLH1 hypermethylation or MSH2 mutation), where MSI-H is the dominant phenotype, and another resulting from nuclear-to-cytosolic shift of MSH3 leading to EMAST being the dominant phenotype. Red cross represents the loss of function.

Contrary to this, complete loss of nuclear MSH3 expression might occur in the context of severe MMR impairment. In this case, MSH3 expression is absent either because of a frameshift mutation of the [A8] microsatellite sequence in *MSH3* gene, which appears after *MLH1* hypermethylation, or due to a mutation in the *MSH2* gene, which leads to degradation of its heterodimeric partners (MSH3 and MSH6) (Figure 2) [33].

An important finding supporting the possible nonmutational MSH3 dysfunction leading to EMAST was the one demonstrating that rectal tumors exhibiting EMAST were also frequently associated with chronic inflammation [34]. Dense immune cell infiltration within these tumors combined with the finding of heterogeneous MSH3 expression in EMAST colorectal cancers indicated that inflammation might have an impact on the functionality of MSH3. Given that the inflammatory tumor microenvironment contains free oxygen radicals from oxidative stress and pro-inflammatory cytokines, follow-up studies have suggested that these factors might be the main drivers of the inflammation-driven loss of nuclear MSH3. In vitro studies have revealed that IL-6 and the downstream JAK/STAT3 signaling pathway, as well as oxidative stress in the form of hydrogen peroxide, can trigger the translocation of MSH3 from nucleus to the cytosol, thereby inducing instability at EMAST loci [32]. In addition, Munakata et al. reported a reduced nuclear expression of MSH3 in the context of increased IL-6 expression in the non-neoplastic epithelium of patients with UC [35]. The explanation behind MSH3 translocation and its possible function in the cytoplasm is still lacking; however, it has been shown that MSH3 contains two cooperating nuclear export signals, both being required for the IL-6 induced MSH3 export, and once IL-6 levels decrease, a single functional nuclear localization signal is responsible for nuclear import of MSH3 [36]. Nevertheless, these findings still have to be confirmed in clinical samples since pro et contra evidence has been presented in the scientific literature [31,32].

### 1.5. Immune Response-Related Features of MSI and EMAST Tumors, Their Clinical Implications, and Relevance for Antitumor Therapy

The identification of MSI has been one of the most significant discoveries for the understanding of tumor–immune relationships. It was hypothesized more than two decades ago that the less aggressive biological behavior of MSI tumors may be explained by their elevated mutational load, which results in higher likelihood of translational frameshifts whose products are recognized by the adaptive immune system, resulting in the pronounced host immune response [37]. This hypothesis was further supported by the observation that the density of infiltrating CD8^+^ T cells is significantly higher in CRC tumors with defective MMR, particularly those harboring a high number of neoantigen-related mutations in coding microsatellites [38]. In addition to that, a subset of dMMR CRCs display high infiltration of activated cytotoxic T cells characterized by interferon-γ production, as well as upregulation of several immune response-related genes, such as granzyme B (*GZMB*), perforin 1 (*PRF1*), and interleukin 21 (*IL21*) [39,40,41]. Such high immunogenicity of dMMR tumors not only helps to explain the favorable prognosis in patients with MSI tumors, but also reflects the higher sensitivity of advanced-stage MSI tumors toward checkpoint inhibition therapy in comparison to MSS tumors. MSI-H tumors also demonstrate higher expression of programmed cell death receptor-1 (PD-1)/programmed cell death ligand-1 (PD-L1) and are usually poorly differentiated, of lower stages, and less invasive tumors [42]. Indeed, it has been established that high levels of microsatellite instability in colorectal tumors are associated with patients’ prolonged progression-free survival and improved clinical benefit of anti-PD1 therapy [43]. Additionally, immune checkpoint inhibitors have shown efficacy across various other MSI tumor types, including gastric, ovarian, and colorectal cancers [44,45]. Recognition of the pronounced immunogenicity of MSI tumors and the expression of multiple counter-inhibitory checkpoints in the background of a robust immune response eventually led to identifying MSI tumors as the optimal target for immunotherapy [45].

Despite this success, even in a group of patients with dMMR tumors, the observed response rates range between 30% and 50%, suggesting the existence of intrinsic resistance mechanisms [43,46]. It is important to note that the presence of immunogenic neoantigens is not the only factor that influences the capacity of T cells to recognize and kill tumor cells. A study conducted by Hu et al. showed that MSI-H CRC tumors basically form two clusters with a difference in the frequency of chromosomal instability, global hypomethylation status, density of M2 macrophages, and survival [47]. Also, a recent study by Sui and colleagues demonstrates that inflammatory conditions in MSI-H CRCs may underlie resistance to immune checkpoint inhibitors through neutrophil leukocyte-associated immunosuppression [48]. In addition, MSI tumors commonly display different levels of genomic heterogeneity which has implications on their response to immunotherapy. Therefore, some authors suggest that a quantitative approach aimed at determining the “intensity of MSI” rather than MSI positive/negative status would be more appropriate for selection of patients for immunotherapy [49].

However, not all tumors with microsatellite instability show such an active intratumoral immune response. Although local inflammation is commonly observed in tumors with dMMR, it appears to be closely related to the underlying genetics. Unlike MSI-H tumors in which defective MMR system is the early event that subsequently leads to the generation of neoantigens and strong activation of adaptive immune system, tumors with EMAST instability appear to be triggered by increased local inflammation that develops during tumorigenesis. The main evidence suggesting that inflammatory microenvironment might be the driver of EMAST instability comes from studies by Devaraj et al. and Lee et al., who observed that among CRCs, EMAST was associated with the presence of chronic inflammation, while its prevalence increased with adenoma to carcinoma progression [34,50].

**Figure 3:**
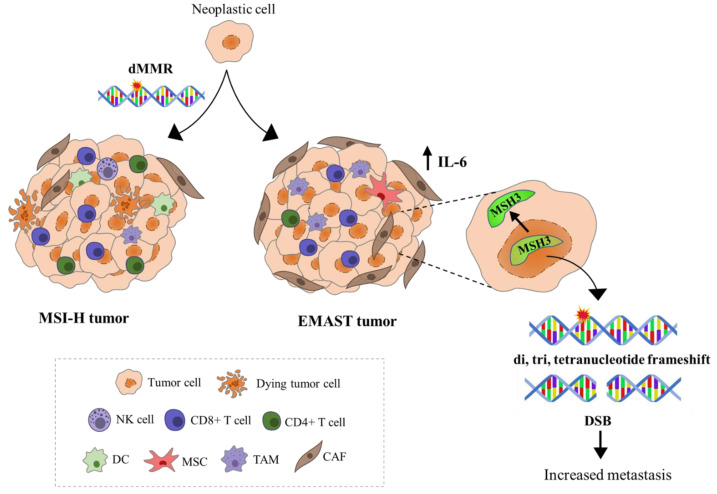
Schematic representation of immunological features of MSI-H and EMAST tumors. Neoantigen recognition in MSI-H tumors triggers the infiltration of CD8+ and CD4+ T cells and natural killer cells (NK cells) into the TME resulting in favorable patient prognosis. EMAST tumors appear to be triggered by increased local inflammation that develops during tumorigenesis. Evidence support the hypothesis that IL-6, which mainly originates from tumor-associated macrophages (TAM), mesenchymal stem cells (MSC), or cancer-associated fibroblasts (CAF), can shift MSH3 protein from the nucleus to the cytosol, allowing accumulation of mutations and double strand breaks. These genetic alterations are believed to influence the tumor behavior, contributing to tumor progression and poorer patient survival.

In the majority of studies, patients with EMAST tumors often exhibit metastasis and have worse prognosis compared to either MSI or MSS tumors [26]. However, a study by Lee et al. revealed that EMAST CRCs show enrichment of CD8+ T cells, but not CD4+ T cells, in the tumor center and surrounding stroma, a feature usually associated with better prognosis in CRC [51]. No studies have directly examined the link between EMAST and tumor mutational load; however, it can be speculated that tumors demonstrating EMAST in the context of a major MMR defect (MSI-H) might be associated with more somatic mutations likely forming more neoantigens, which can consequently trigger the activation of adaptive immune response. Chen et al. have shown that patients with MSI-H/EMAST CRCs have longer survival compared to those with only MSI-H tumors, suggesting that the survival benefit possibly arises from an additive effect of EMAST [52]. Studies examining the immune milieu of EMAST tumors arising from isolated MSH3 dysfunction are scarce. Figure 3 is an attempt to present immunological features of tumors exhibiting EMAST in the context of MSI-H and tumors in which only EMAST is present. A more detailed insight in the immune microenvironment of tumors with different EMAST etiologies should be imperative of future studies as its presence can signify increased risk for tumor progression as well as the possibility for therapeutic interventions.

In spite of the fact that impaired MSH3 function could be triggered by its nuclear-to-cytosolic translocation, a process possibly mediated by pro-inflammatory IL-6 and oxidative stress, the precise mechanism linking inflammation and EMAST originating from the heterogeneous loss of MSH3 nuclear expression is still being investigated. Studies aimed at directly determining the association between EMAST, IL-6, and oxidative stress have failed to give the final conclusion; however, if we consider EMAST in the context of chronic inflammation, at least in the colon, more evidence is mounting. Indeed, EMAST was detected in pre-neoplastic or non-neoplastic tissue of ulcerative colitis patients with rising frequency from tissues without neoplasia to tissues with dysplasia and further on to colorectal tumors arising from ulcerative colitis background [35]. Also, EMAST was more frequently observed in ulcerated tumors compared to sessile and protruded tumors [51].

The source of inflammation triggering EMAST has not been investigated but could involve alterations in microbiota composition and its metabolic products, as well as disruption of intestinal barrier by the tumor process itself [53]. For instance, an opportunistic pathogen *Fusobacterium nucleatum* is particularly abundant in the intestinal microbiota of MSI-H CRC patients [54,55] and can exert its tumor-promoting effects through several mechanisms, including CRC cell growth and proliferation [56,57], promoting inflammation and restraining antitumor activity [58,59]. *F. nucleatum* infection is associated with a specific pro-inflammatory signature in tumors characterized by expansion of myeloid-derived suppressor cells and expression of several pro-inflammatory mediators, such as IL-6 and IL-8 [59]. Based on these findings, Okita et al. suggested that moderate loads of *F. nucleatum* might also contribute to the generation of EMAST in tumors and promote CRC tumorigenesis by inducing DNA damage [60].

Although MSI tumors have traditionally been the primary targets for immunotherapy, results from the study conducted by Chen et al. suggest that CRCs exhibiting both MSI-H and EMAST might be more suitable targets [52]. If EMAST tumors share the characteristics of MSI-H tumors, they may also exhibit enhanced response to immunotherapy. However, with the lack of major MMR defect leading to MSI-H, it could be speculated that EMAST tumors do not exhibit a hypermutated phenotype, which makes them less prone to activate adaptive immune system and less likely to express immune checkpoint inhibitors, which in turn might lead to different response to the immunotherapy. Nonetheless, recent studies have unveiled promising prospects for response in microsatellite stable (MSS) tumors, which represent the vast majority (95%) of patients with mCRC [61]. Since EMAST tumors display higher density of TILs in comparison to EMAST-negative tumors but are also often associated with metastatic progression and poor survival in patients, discovering innovative therapeutic interventions, particularly those involving the immune system, holds promise for the treatment of patients with metastatic disease.

### 1.6. Challenges of MSI and EMAST Detection

As the awareness of other possible outcomes of defective MMR (dMMR) has evolved, studies examining the microsatellite instability at tetranucleotides were beginning to reappear, and the term EMAST was introduced in the scientific community. No consensus has been established for EMAST detection, but a panel of five tetranucleotide markers (MYCL1, D9S242, D20S85, D8S321 and D20S82), sometimes expanded by additional tetranucleotides, is most commonly used [34].

Although EMAST’s name suggests, that only changes at tetranucleotide microsatellite loci are involved, MutSβ heterodimer detects frameshifts in microsatellite sequences ranging from two to 13 nucleotides, suggesting that changes in all but mononucleotide repeats might occur associated with EMAST. In contrast, MutSα recognizes single mismatches and frameshifts at dinucleotide repeats, meaning that MSI affects mainly these loci [18]. It seems, therefore, that, at least theoretically, the main difference between MSI and EMAST is that the latter encompasses frameshifts at di-, tri-, tetra and longer microsatellite repeats with the exclusion of mononucleotides [18].

However, currently used MSI and EMAST screening panels fail to detect this difference. EMAST-positive tumors with isolated MSH3 dysfunction by definition cannot present with mononucleotide frameshifts; however, EMAST analysis is not included in the conventional Bethesda panel as it has not been considered important for diagnostic, prognostic, or therapeutic purposes [8]. The same is true for the assessment of mismatch repair defects by immunohistochemistry at the protein level since MSH3 protein expression is not included in the pathologists’ guidelines for dMMR detection in histopathological samples [62].

Thus, in studies using conventional methodology for MSI/dMMR assessment, some tumors classified as MSI tumors might in fact be MSI/EMAST tumors. On the other hand, some MSI-L or even MSI-H (if two markers of the Bethesda panel are affected) tumors may in fact have only dinucleotide frameshifts, a defect which can be indicative of isolated MSH3 dysfunction, and thus share more similarities with EMAST than the MSI tumor phenotype [18].

Since there is no consensus on the polymorphic markers used for EMAST detection, most studies assess only the status at tetranucleotide loci and are ignorant of what is happening at mono- and dinucleotides. As we have described previously, EMAST can also arise as a secondary event in MSI unstable tumors, in which case tumors display mono- along with di- and tetranucleotide frameshifts. It is important to note that defects leading to these comprehensive changes are mostly due to MLH1 and MSH2 dysfunction, which are highly destabilizing to the tumor genome and, thus, these MSI/EMAST tumors display different phenotypic characteristics with respect to tumors with only EMAST instability [4]. Again, this is missed in most studies examining only the EMAST status.

As studies examining both MSI and EMAST status of tumors are rare, it is clear that some tumors with dinucleotide instability considered as MSI are in fact a result of isolated MSH3 dysfunction and, thus, display characteristics of the EMAST phenotype solely. Similarly, some of EMAST tumors also harbor mononucleotide frameshifts and, thus, are primarily a result of MLH1, MSH2, or PMS2 defect and mostly display phenotypic characteristics of MSI-H rather than EMAST tumors.

### 1.7. Why Is the Isolated MSH3 Dysfunction so Difficult to Pinpoint?

Several factors have contributed to rather late recognition of isolated MSH3 dysfunction. Chronologically speaking, microsatellite instability was first examined in the context of a hereditary form of colorectal cancer, Lynch syndrome. However, until now, there have been no identified germline mutations in the *MSH3* gene associated to Lynch syndrome. Next, it was observed that CRC tumors in which MLH1 dysfunction is present (either hereditary or sporadic) in a fraction of tumors (approximately 30% of MSI-H sporadic CRCs or 5% of all sporadic CRCs) lead to subsequent *MSH3* mutation and inactivation. Nevertheless, the contribution of MSH3 dysfunction to the existing MSI phenotype was not observed [63]. *MSH3* gene silencing via promoter methylation has been reported in sporadic gastric cancer; however the subsequent consequences of this event on tumorigenesis have not been clarified [64]. Furthermore, Carethers et al. reported that *MSH3* promoter hypermethylation is not the mechanism of its inactivation and neither are mutations since they occur rarely in colorectal tumors [63]. This led to a subsequent perception that MSH3 is not so important in tumorigenesis, at least in the context of colorectal cancer, in which it was mostly investigated. Furthermore, until recently, no germline mutations of *MSH3* have been reported [65]. Interestingly, in the study in which bialellic *MSH3* germline mutations in two unrelated individuals with unexplained adenomatous polyposis (later named oligopolyposis) were reported, the authors have examined the microsatellite status of di- and tetranucleotide markers in both normal and tumor tissue and EMAST was detected in both. Furthermore, immunohistochemical analysis showed an almost complete absence of MSH3 protein in cells, and a complete loss of its presence in the nucleus in both normal and tumor tissues of these individuals. Nevertheless, MSH3 mutations remain extremely rare in contrast to the quite frequent detection of EMAST in tumors.

Major progress towards the elucidation of mechanisms potentially explaining isolated MSH3 dysfunction was made by Tseng-Rogenski et al. who demonstrated that MSH3 inactivation might be the result of a nuclear-to-cytoplasmic shift, triggered by IL-6 or oxidative stress [31,32]. But is the loss of nuclear MSH3 staining due to its translocation to the cytosol observed in clinical tumor samples? The final conclusion has not been reached due to conflicting findings of only a handful of studies performed in CRC [23,50,66,67,68]. In addition to the fact that MSH3 IHC shows a heterogeneous nuclear-staining pattern, which requires counting of negative nuclei, the definition of what constitutes MSI and EMAST differs from study to study. Here are some examples of issues that have to be resolved before any final conclusions can be reached.

In a study by Haugen et al., the authors claim that MSH3 deficiency is the cause of EMAST [23]. They used the Bethesda panel with the addition of two dinucleotide markers for MSI, and seven tetranucleotide markers for EMAST assessment. Tumors were considered MSI-H if three or more markers were affected. Tumors with one or two unstable markers were considered as MSI-L. EMAST was defined if one or more markers were affected by instability. In their study, both MSI-H and MSI-L tumors were also positive for EMAST. In addition, samples denoted MSS/EMAST were also identified. All MSI-L tumors were unstable only at dinucleotide loci. Moreover, consistent with the trend in the field at that time, authors also speculated that the observed MSS/EMAST might in fact be MSI-L/EMAST, if additional dinucleotide markers were to be included. The loss of MSH3 was determined by IHC as a percentage of negative nuclei. In their study, EMAST-positive tumors had 31.5% of negative nuclei, while EMAST-negative tumors had only 8.4% of negative nuclei. Tumors with more than 15% of negative nuclei were considered as MSH3-negative. The observed presence of MSH3 positive nuclei in EMAST tumors was explained by possible reversibility of MSH3 localization. The conclusion of the study was that the loss of MSH3 is associated with EMAST.

On the other hand, in the study by Watson et al., which claimed that loss of MSH3 is not associated with EMAST, MSI was defined using only mononucleotide markers and MSI-H was declared if two or more out of five markers tested positive for instability [67]. Similarly, EMAST was noted if two or more tetranucleotide markers tested positive for instability. Thus, instability at one EMAST locus, which some authors count as low EMAST, was ignored and these tumors were considered stable. In addition, most of the samples in this study have proven to be MSI-H/EMAST. On the other hand, MSH3 IHC was classified according to groups consisting of 1%, 5%, 10%, 25%, and 50% of MSH3 nuclear loss. No association between MSH3 loss and EMAST was observed with any of these groups.

These two opposing examples clearly demonstrate that MSH3 IHC/EMAST studies again suffer from arbitrary cut-offs both in terms of IHC evaluation as well as EMAST definition. Moreover, most studies only focus on possible association between the loss of nuclear MSH3 protein staining ignoring different mechanisms leading to it (MSH3 frameshift vs. MSH3 translocation) with possibly different IHC patterns as reported by Haugen et al. [23]. All these issues still render MSH3 IHC problematic for any routine clinical application [68].

However, these conflicting findings might not be in contradiction with the proposed mechanism of the MSH3 nucleus-to-cytosol translocation after all. Contrary to in vitro experiments, clinical samples represent a snapshot of a tumor “frozen in time” of its collection. Since the proposed mechanism of MSH3 inactivation is by its nature probably reversible, as suggested by the existence of nuclear localization and export signals [36], the change in MSH3 localization might be long gone by the time the tumor sample is collected, even though the damage it causes remains. In fact, studies which were not able to confirm that MSH3 translocation is associated with EMAST at the genomic level might in fact suffer from this exact problem of MSH3 dysfunction being a transitory cause, while EMAST is a permanent consequence. Moreover, most studies, again, do not discriminate between EMAST as a primary event due to isolated MSH3 dysfunction, or secondary event due to MSI. Additionally, tumor evolution might in different tumor phases favor a subset of cells in which MSH3 is no longer present or relevant.

More indirect evidence about different mechanisms of MSH3 loss of function giving rise to EMAST is emerging. For instance, in the study by Meessen et al., in sporadic colorectal cancer in tumors with one to two markers positive for EMAST, downregulation of *MSH3* mRNA was not detected, while in tumors with three to five markers positive for EMAST (which often coincides with MSI-H), *MSH3* mRNA expression was downregulated [69]. Both findings are consistent with two different mechanisms proposed for EMAST generation. No change in *MSH3* mRNA expression is consistent with the MSH3 protein nuclear-to-cytosol shift [31,32], while downregulation of *MSH3* mRNA expression is consistent with the mutational inactivation of the *MSH3* gene as a result of, for instance, hypermethylation of *MLH1* promoter [33].

Moreover, Meessen et al. also examined *MSH3* gene alterations and expression in approximately 3000 colorectal tumors from the publicly available database cBioPortal for Cancer Genomics. According to their results, *MSH3* mutations were present in 1–6% of samples depending upon the analyzed cohort of patients. On the other hand, copy number of alterations of the *MSH3* gene were detected in about 32% of CRCs and these changes mostly did not overlap with MSI-H tumors, consistent with the idea that MSI does not coincide with CIN [69]. These results also point to a possible association of EMAST/MSI-L (or non MSI-H) with CIN as reported by Shin at al. [70]). Similar findings were reported for a publicly available cohort of pancreatic cancers also examined in this study. In both colorectal and pancreatic cancer, no hypermethylation of *MSH3* promoter was detected [70].

### 1.8. Is EMAST a Distinctive Entity Separate from MSI and MSS and Do We Need to Discriminate between Different EMAST Etiologies with Respect to Patients’ Prognosis and Therapy?

Although much progress has been achieved in detection and treatment of early stages of most common cancers, disease recurrence with local invasion or systemic spread of metastasis still remains a major problem for advanced cancers. Markers that could predict prognosis and/or determine the best choice of targeted therapy for each patient according to the (epi)genetic changes within a tumor still remain a Holy Grail of personalized oncology. One of the reasons is certainly the intrinsic heterogeneity of cancerous and even stromal cells within a tumor. Defects of DNA mismatch repair contribute to the speed-up of molecular evolution of tumor cells. Creation of neoantigens as a result of defective MMR is deemed beneficial for patients since it mobilizes their immune defense. In addition, it also has beneficial effects for the response to immunotherapy. But is every microsatellite instability made equal? Do MSI and/or EMAST have the same influence on tumor evolution and, thus, patient prognosis? Moreover, one can speculate that EMAST might perhaps act as a modifying factor in a subset of these tumors.

At present, these questions seem to be unresolved partly due to the design of most studies examining the influence of MSI or EMAST on patients’ outcomes and response to therapy. Most studies have only evaluated the tumor MSI status, while studies evaluating the influence of EMAST often do not examine the MSI status of analyzed tumors. On the other hand, it has become increasingly clear that predicting the response of patients to immunotherapy solely based on one biomarker, for instance the MSI-H/dMMR status in the case of anti-PD-1 immunotherapy in metastatic and unresectable refractory colorectal tumors, was oversimplistic and overoptimistic. Studies show that even with that type of patient, preselection response rate to checkpoint inhibitors is between 30% and 40% [43,71,72].

One can argue that MSI-H can clearly be distinguished from MSI-L and MSS tumors due to hypermutable molecular profiles caused by major dMMR (mostly of MLH1 or MSH2 origin), which also lead to different clinicopathological characteristics of these tumors [73]. The difference between MSI-L and MSS is less obvious, and some authors have defined them as two separate entities, while other have claimed that this difference does not exist [74,75].

Most, if not all, early studies were agnostic to the status of microsatellite instability at tetranucleotides. In addition to analyzing the Bethesda panel, certain studies that identified EMAST as a distinct form of instability have also incorporated the assessment of the tetranucleotide status in the analyzed samples.

When EMAST was recognized as a specific type of instability, some studies have, in addition to the Bethesda panel,,also included the assessment of tetranucleotide status in the analyzed samples. As early as 1995, in a study examining MSI and EMAST in colorectal cancer, it has been shown that most EMAST-positive tumors are also MSI-H tumors [22]. By using the 10 tetranucleotide markers for EMAST and Bethesda panel for MSI evaluation, authors reported that only 1.3% of EMAST-positive tumors were not associated with MSI-H. It could be expected that tumors in which MSI-H was present also exhibit high levels of instability at tetranucleotide loci, which is consistent with the previously noted global impact of dMMR caused by impairment of major MMR genes. Nevertheless, EMAST was also detected in both MSI-L and MSS samples [23,24]. Haugen et al. showed that in colorectal cancer, MSH3 loss of function can result in MSI-L (at dinucleotide loci from the Bethesda panel) and EMAST. Moreover, these tumors differ from MSI-H and MSS tumors, constituting a kind of “in between“ moderate type of microsatellite instability (MSI-M) [23]. Again, Garcia et al. showed that in CRC, stage II or III patients whose tumors were classified as MSI-L with or without EMAST had shorter recurrence-free survival when compared to patients with either MSS or MSI-H tumors [26]. This status was also an independent predictor of recurrent distant metastasis. They also recognized that the etiologies of MSI-H/EMAST and MSI-L/EMAST are different. The former results mostly from MLH1 or MSH2 dysfunction, while the latter results from MSH3 dysfunction. Chen et al. have shown that patients with MSI-H/EMAST colorectal tumors had higher tumor mutation burden and longer survival compared to MSI-H tumors without EMAST and thus potentially could also have a better response to immunotherapy [52]. Nevertheless, no clinical study has been performed to test this hypothesis.

In recent years evidence of MSI-L or EMAST/non-MSI-H as a poor prognostic marker in CRC is mounting as is the idea of their common etiology [23,26,69,76]. In proof of the idea that an isolated MSH3 dysfunction may lead to different pathways of tumorigenesis are studies in which the presence of EMAST was examined in the context of chronic inflammation. In a study by Munakata et al., a reduced nuclear MSH3 expression in the context of increased IL-6 expression was reported in the non-neoplastic epithelium of ulcerative colitis patients [35]. In addition, EMAST was detected in the same samples. Moreover, CRC tumors with UC background had a higher frequency of EMAST compared to early-stage sporadic CRCs. In an earlier study, Koi et al. showed that there is not much difference in the presence of EMAST due to isolated dysfunction of MSH3 in metastasis compared to primary CRC tumors. However, loss of heterozygosity was more frequent in metastases [25]. These findings suggest that the repair of double-stranded brakes might also influence the phenotype and characteristics of EMAST tumors.

If differentiating between two possible mechanisms of EMAST generation is not irrelevant with respect to patient outcome and response to therapy, then this mechanistic difference has to be examined for each patient. However, studies designed to discriminate between these two mechanisms are scarce. One can only speculate that perhaps tumors with EMAST as a result of an isolated MSH3 dysfunction could respond differently to immunotherapy, due to their lesser immunogenicity and different mutational spectra, compared to MSI-H/EMAST tumors resulting from dysfunction of other MMR genes.

Studies in other types of cancer aiming to resolve the role of MSI and EMAST in tumorigenesis are scarce. In the study aiming to clarify whether EMAST is a distinct type of microsatellite instability with potential clinical impact in gastric cancer, Herz et al., in addition to using the standard Bethesda panel for initial MSI screening, also used another panel with three additional mononucleotides [77]. The purpose of this second panel was to distinguish between MSI-H and MSI-L in samples in which, by using the Bethesda panel, only dinucleotides were affected. EMAST was evaluated utilizing the commonly used set of five tetranucleotide markers and defined by using the two degrees of stringency (instability at two or three tetranucleotide markers). No difference in overall patient survival and response to neoadjuvant CTx between EMAST-positive and negative tumors was observed. This suggests that subgroup of gastric tumors positive only for EMAST does not present with distinctive clinico-pathological characteristics compared to EMAST-negative tumors.

Interestingly, among the first studies recognizing EMAST as a separate entity was a study by Ahrendt et al. performed in non-small cell lung cancer (NSCLC). Among 88 analyzed tumors, 31 (35%) were EMAST positive for at least one of 13 analyzed markers, while none were MSI positive as tested by using the conventional Bethesda panel [78]. Thus, in this study, EMAST was recognized as a separate entity; moreover, these tumors also had higher frequency of *p53* mutations, and their phenotype was different to what is expected from MSI tumors. In a later study by Woenckhaus et al., MMR status was examined by immunohistochemistry, while EMAST was examined using the di-, tetra- and pentanucleotide markers [79]. In essence, all analyzed samples were MMR proficient, while EMAST was found in 19.1% of analyzed NSCLCs. The presence of EMAST was associated with lymph node metastasis, squamous differentiation, and LOH of *p53*, consistent with the previous study. In a later study performed in NSCLC by Arai et al., the presence of EMAST was independent of the MSI status of the tumor [80]. Thus, it appears that in NSCLC, EMAST defines a separate subgroup of tumors. Similar findings have been reported for pancreatic ductal adenocarcinoma with no difference in clinico-pathological characteristics and survival of patients with EMAST-positive or negative status [81].

In a study evaluating the occurrence of EMAST in prostate cancer by Burger et al., MSI status was determined using the consensus Bethesda panel in addition to BAT40 mononucleotide marker, while EMAST was evaluated using a panel of 10 tetranucleotide markers [82]. Interestingly only 5% of analyzed tumors showed EMAST, while 18.5% of tumors showed LOH at some of the analyzed EMAST markers. Nevertheless, in this study the frequency of MSI was also somewhat low (7.6%) and only one tumor was identified as MSI-H, while one EMAST-positive tumor was also MSI-L. The same group also examined the status of bladder cancer with respect to MSI and EMAST using the consensus Bethesda panel and additional 12 tetranucleotide markers. Again, the frequency of EMAST was low (8.5%), while LOH at EMAST markers was present in 5.3% tumors, and MSI was detected in only one sample (0.9%) [82]. In a study by Singer et al. examining MSI and EMAST in ovarian cancers, MSI was tested using the conventional Bethesda panel and EMAST was evaluated using six tetranucleotide markers [83]. Tumors exhibiting MSI-H were mostly EMAST negative and EMAST was detected only in advanced tumors.

Some attempts have also been made to unify MSI-L (based on dinucleotide frameshifts) and EMAST found in sporadic colorectal, endometrial, ovarian, lung, melanoma, pancreatic, gastric, and bladder tumors, under the term “alternative MSI forms”; however, this terminology was not embraced by the wider scientific community [84]. Nevertheless, in this comprehensive review, several possible explanations of “alternative MSI forms” are given. Most classifications define MSS tumors as those with no microsatellite instability; however, some others allow for the “in between group” with up to 30% of instability at selected microsatellite loci as a result of stochastic mutational events. The authors start with the idea that tumors classified according to the Bethesda and similar panels consisting of mono- and dinucleotide markers fall in two major groups: MSI-H and MSS tumors. Next, Hile et al. offer several possible causations for, as they call them, “alternative MSI forms” observed in tumors, including experimental artefacts, spontaneous mutations at microsatellite loci, moderate defects in DNA repair and/or replication, damage-induced changes, and finally, a possibility that MSI-L at dinucleotides and EMAST are distinct genetic entities [84]. Most of the argumentation is still valid (for details please refer to the source); however neither these arguments, nor the most recent studies involving the NGS data, have given the final answer to the proposed question.

## 2. Conclusions

Most of the conflicting results regarding the differences between MSI and EMAST and whether EMAST more resembles MSS tumors derive from the fact that studies rarely examine both types of instability in the same sample as well as from inconsistent definitions of EMAST. Some studies define it as a distinct phenomenon arising from the isolated MSH3 dysfunction, which is not a secondary event to MSI and, thus, the samples are often defined as EMAST positive with the absence of MSI-H. However, although MSI-H mostly includes instability at mononucleotides, it must be noted that samples positive for instability at two dinucleotide loci used in the Bethesda panel, of which changes can hypothetically also arise from the isolated MSH3 dysfunction, are also classified as MSI-H. Similarly, in a number of other studies, MSI-L, which may or may not include mononucleotides, is often grouped with EMAST, although the etiology of EMAST in these samples is sometimes a result of isolated MSH3 dysfunction and sometimes (when mononucleotides are involved) is a secondary event to MSI. This clearly shows that when MSI and EMAST or MSI-H/MSI-L terminology is used, it creates an overlap or blurs the within-sample groups regarding the true etiology of these changes.

So, what are the possible solutions of how to overcome the overlap or blurring between EMAST and MSS or EMAST and MSI?

Some authors have proposed the use of other terminologies, such as alternative forms of MSI [84] or inflammation-associated microsatellite alterations (IAMA) when addressing EMAST arising from an isolated MSH3 dysfunction [18]. However, this has not been widely accepted in the scientific community, which still mostly perpetuates the MSI/EMAST terminology often without the clear definition of what each term actually means in terms of the etiology of microsatellite instability. Some authors acknowledge that each of these classifications is in fact an approximation and state that most MSI-H samples are those harboring mononucleotide frameshifts, while most MSI-L samples show mostly changes at dinucleotides rather than mononucleotides [18,26,84]. Although a step back from these commonly used terms would be useful, any distancing also results in nonevocativeness of the proposed terminology or impractical descriptive definition of samples, which include EMAST and microsatellite instability at dinucleotides, or its absence in mononucleotides. Some rare studies approach this problem by including more microsatellite markers until all samples are unambiguously classified; nevertheless, adding more markers still does not solve the causational etiology of EMAST as primary isolated change, or secondary to MSI [23,24,26].

Another possible way to overcome this problem of overlap or blurring between EMAST and MSI or EMAST and MSS tumors is to include some other defining features in addition to the analysis of frameshifts at microsatellite loci. Most obvious is the immunohistochemical analysis of MMR proteins, including MSH3, which is not commonly assessed for dMMR detection at the protein level in clinical settings. Although this approach might seem useful to further entangle the etiology of microsatellite instability, due to the reported disconcordance between IHC and analysis of microsatellite instability at the genomic level, a small fraction of samples will still potentially be misclassified [85,86]. Adding to this, it is also worth noting that results of IHC are not always unambiguous, and this is especially true for proteins MSH2 or MSH3, which mostly display a heterogeneous nuclear-staining pattern with respect to their loss of function [23,50,87]. For instance, some authors have noted that MSH3 dysfunction secondary to mutation caused by MLH1 or MSH2 dysfunction often results in total loss of MSH3 expression, while isolated MSH3 dysfunction gives a heterogeneous nuclear-staining pattern; however, this has to be examined and verified in much larger cohorts [33].

The proposition that MSH3 dysfunction in addition to EMAST generates double-stranded breaks is not relevant for determination of EMAST as a primary event or secondary to MSI since in both cases the resulting MSH3 would be associated with double-stranded breaks [88].

If EMAST, as a result of isolated MSH3 dysfunction, and MSI are truly different entities, then perhaps a mutational spectra of these tumors might also differ. Thus far, there are few studies examining the mutational spectra of tumors with an established MSI and EMAST status, which mostly focus on the possible additive effect of EMAST to MSI changes in these tumors without discriminating between the two different EMAST etiologies [52,89]. In a study by Chen et al., MSI-H/EMAST tumors had different clinicopathological and mutational features, so the authors concluded that this subgroup of patients might respond better to immunotherapy compared with patients whose tumors are only MSI-H without the additional EMAST [52]. Defining a specific mutation profile of EMAST tumors arising secondary to MSI or as a primary event due to isolated MSH3 dysfunction would perhaps also help in better stratification of patients in terms of their prognosis and response to therapy. On the other hand, as we have mentioned previously, inclusion of CIN analysis might also aid in further stratification of patients.

In recent years, a number of studies employing NGS in the detection of MSI in cancers have emerged. Often in conjunction with NGS, a tumor-mutational burden or specific mutations within a tumor are also evaluated [90]. This approach has led to further molecular profiling of MSI tumors with possible applications to patient prognosis and response to therapy. Nevertheless, no consensual clinically validated NGS protocol, which would fulfil the idea of personalized approach to each patient, has been established. Although NGS has clearly huge possibilities in the clinical settings, the technology is still expensive and requires a high degree of expertise, which is still not available in most clinical laboratories.

Another avenue worth exploring are immunological differences among MSS, MSI, and EMAST tumors, but, as was stressed previously, finding those differences is extremely demanding and requires a significant level of expertise. Thus, studies examining this aspect of possible differences between MSS, MSI, and EMAST tumors are extremely rare, but necessary.

Finally, if discriminating between different types of microsatellite instability proves to be relevant for patient prognosis and therapy, the proposed screening methods have to be manageable in the settings of clinical pathology laboratories.

## Data Availability

Not applicable.
